# Upcycling Post-Consumer Paint Pail Plastic Waste

**DOI:** 10.3390/polym16182631

**Published:** 2024-09-18

**Authors:** Rajkamal Balu, Swati Sharma, Rachael Roberts, Jitraporn Vongsvivut, Namita Roy Choudhury

**Affiliations:** 1 Chemical and Environmental Engineering, School of Engineering, STEM College, RMIT University, Melbourne, VIC 3000, Australia; 2 ARC Industrial Transformation Research Hub for Transformation of Reclaimed Waste into Engineered Materials and Solutions for a Circular Economy (TREMS), RMIT University, Melbourne, VIC 3000, Australia; 3 Paintback Limited, South West Suite, Level 3, 717 Bourke St, Docklands, VIC 3008, Australia; 4 ANSTO—Australian Synchrotron, 800 Blackburn Road, Clayton, VIC 3168, Australia; jitrapov@ansto.gov.au

**Keywords:** recycled polypropylene, waste wool fiber, melt processing, composites, thermal analysis, rheology, mechanical properties

## Abstract

The need for ending plastic waste and creating a circular economy has prompted significant interest in developing a new family of composite materials through recycling and recovery of waste resources (including bio-sourced materials). In this work, a family of natural fiber-reinforced plastic composites has been developed from paint pail waste recycled polypropylene (rPP) and waste wool fibers of different diameter and aspect ratio. Composites were fabricated by melt processing using polypropylene-graft-maleic anhydride as a compatibilizer. The internal morphology, interfacial and thermal characteristics, viscoelastic behavior, water sorption/wettability, and mechanical properties of composites were studied using electron microscopy, high-resolution synchrotron Fourier transform infrared microspectroscopy, thermal analysis, rheology, immersion test, contact angle measurement, tensile test and flexural test. The composite matrix exhibited an internal morphology of coalescent micro-droplets due to the presence of polyethylene and dry paint in the rPP phase. In general, the rheological and mechanical properties of the composites comprising higher-aspect-ratio (lower diameter) fibers exhibited relatively superior performance. About an 18% increase in tensile strength and a 39% increase in flexural strength were measured for composites with an optimal fiber loading of 10 wt.%. Interfacial debonding and fiber pull-out were observed as the main failure mechanism of the composites. The developed composites have potential for applications in automotive, decking, and building industries.

## 1. Introduction

Plastic and textile waste pollution is a global problem. Approximately 400 million tonnes of plastic and 92 million tonnes of textile wastes are generated globally each year, of which only 9% plastic and 12% textile waste are recycled, while the rest end up as landfill or incinerated [1,2]. Waste valorization (i.e., converting waste into value-added products) is a promising solution for the rising demand of plastic and textile waste management and creating a sustainable, and circular, economy [3,4]. Waste materials destined for landfill can be redirected and repurposed in this way for light-weight value-added applications and reducing dependence on petrochemical-based materials. However, the valorization of plastic waste, such as plastic pails (largely polypropylene) is quite challenging due to dry paint contamination, which cannot be removed by currently established industrial mechanical recycling processes and could potentially affect the morphology and property of the product. Although organic solvent [5]- and laser technology [6]-based removal of paint from plastic has been proposed, their application at an industrial scale is limited by the cost and hazards involved. Moreover, the existing sorting and recycling technologies do not yield pure polypropylene, and typically contain some fraction of polyethylene products, which affect their morphology and properties [7]. Therefore, the valorization of recycled plastic containing dry paint and polyethylene as contamination in the formulation of composite development could be a sustainable solution for managing paint pail waste and textile waste.

Different approaches for the valorization of plastic waste have been reported in the literature, where development of composite materials with enhanced properties could be advantageous in utilizing the current infrastructure of polymer processing industries for product fabrication [3]. Over the past two decades, research on fiber-reinforced polymer composites has shown significant improvement in their thermal stability, and mechanical and sound absorption properties [8]. Particularly, natural fiber-reinforced polymer composites (NFRPCs) have gained increasing attention as sustainable materials for emerging applications due to their light weight, low cost, low environmental impact, and notable mechanical properties [9]. Moreover, the NFRPCs could be recycled approximately 4–6 times before they lose their properties [10]. Several plant-based natural fibers including spruce [11], kenaf [12], hemp [13], cellulose [14], pineapple crown [15], and jute [16] fibers have been reported (in the range of 5–50% loading) for reinforcing recycled polypropylene, which has exhibited improvement in tensile, flexural, and compressive strengths. However, only a few studies have been reported for animal-based natural fibers, such as silk [17], wool [18], and their mixed fibers [19], for reinforcing virgin polypropylene. Silk and wool fibers largely used in literature studies were obtained as pre-consumer products or industry wastes, such as sliver [20] and selvedge [21], which has relatively superior quality, whereas the application of post-consumer textile waste fibers in the development of recycled polypropylene-based composites remains under-examined.

Wool with its excellent thermal insulation property is considered as one of the most utilized and recycled animal fibers in the world [22], where around 22,000 tonnes per annum of wool rags is currently recycled in Prato, Italy, for manufacturing recycled wool blend sweaters [23]. In general, animal wool fibers consist of 39.6 mol % non-polar, 40.2 mol % uncharged polar, and 20.2 mol % charged polar amino acid residues, including two sulfur-containing residues (11.2 mol % cysteine, and 0.5 mol % methionine; with 5 wt.% sulfur [24]), and exhibit a water contact angle of 78.2 ± 5.9° [25], which could increase to ~131° when knit into fabric and further treatments [26]. The molecular structure of wool fibers consists of α-helical monomer domains, which form a coiled-coil dimer structure and further organize into protofilaments, protofibrils, and microfibrils. Natural wool fibers exhibit an elastic modulus, ultimate strength, and breaking strain in the range of 2.3–3.4 GPa, 120–174 MPa, and 25–35%, respectively [27]. Considering all these attributes, in this work, we focus on the development of textile waste wool biofiber-reinforced paint pail waste recycled polypropylene composites, and investigate their structural, interfacial interaction, thermal, rheological, and mechanical properties to develop a structure-property relationship.

## 2. Materials and Methods

### 2.1. Materials

Flakes and fibers (1-to-2-inch size) of rPP (collected from paint pail waste, and subsequently washed, sorted, and shredded) with some dry paint contamination were provided by Paintback Limited, Melbourne, VIC, Australia. The rPP was further shredded and sieved using a 6 mm sieve for polymer processing. The two types of textile waste wool fibers (WF1—light blue color, and WF2—light pink color; fiber dimensions provided in Section 3) used in this work were supplied by Paintback Limited, Melbourne, VIC, Australia, and the fibers were used as received without any modification. Polypropylene-graft-maleic anhydride (PP-g-MA) with 8–10 wt.% maleic anhydride was procured from Sigma-Aldrich, Melbourne, VIC, Australia.

### 2.2. Fabrication of Composites

**Figure 1:**
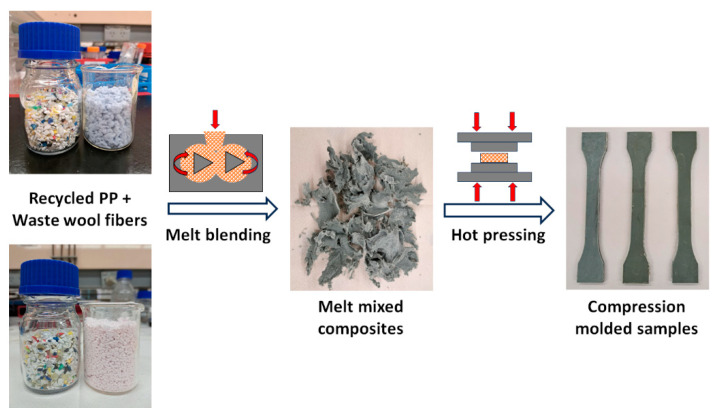
A schematic of the composite fabrication process. The red arrows indicate the direction of mechanical movement of equipment (left—mixer; right—press) parts.

The wool fibers were pre-dried at 75 °C for 24 h in a laboratory vacuum oven to remove any moisture. Samples were fabricated by first melt blending rPP (at 175 °C) in HAAKE^TM^ Rheomix OS Lab Mixer (equipped with roller rotors and operated at 20 rpm screw speed) connected to HAAKE^TM^ PolyLab^TM^ OS System (Thermo Fisher Scientific Australia Pty Ltd., Scoresby, VIC, Australia), followed by the addition of a mixture of PP-g-MA and dry wool fibers (WF1 and WF2) and further mixing for a total time of 15 min. The melt-mixed samples were then cooled down to room temperature, cut into smaller pieces, and compression-molded (at 180 °C with 150 psi pressure and curing time of 2 min) to required specimen dimensions. A schematic of the composite fabrication process is shown in Figure 1, and the compositions of fabricated samples are given in Table 1. A compatibilizer (PP-g-MA) concentration of 5 wt.% was used in this study, as it has been previously reported to be suitable for fabricating polypropylene/natural fiber composites with up to 40 wt.% fiber loading [18,28]. For rheology and surface roughness experiments, disk-shaped specimens (25 mm diameter and 2 mm thickness) were compression-molded. For mechanical property measurements, dumbbell (Type I specimens with a thickness of 3.2 mm, overall length of 165 mm, width of 13 mm, and gauge length of 50 mm)- and rectangular (a thickness of 3.2 mm, width of 12.7 mm, and length of 127 mm)-shaped samples were compression-molded according to ASTM (D638 and D790) testing standards. The cross-section of composite samples (prepared for mechanical testing) was microtomed to 60 µm thick films for a synchrotron Fourier transform infrared (FTIR) microspectroscopy analysis.

### 2.3. Characterization

The functional groups of wool fibers, rPP, and PP-g-MA were analyzed using a Spectrum 100 FTIR spectrometer (PerkinElmer Pty Ltd., Glen Waverley, VIC, Australia) equipped with an attenuated total reflectance (ATR) accessory. The spectra were collected in the wavenumber range 4000–750 cm^−1^. The surface morphology of the received wool fibers, and tensile fracture cross-section area of compression-molded samples, was analyzed using a Quanta 200 scanning electron microscope (FEI Technologies Inc., Hillsboro, OR, USA). The samples were coated with 10 nm thick iridium coating prior to the analysis. The spatially resolved distributions of the chemical functional groups in composite samples were analyzed using the synchrotron macro-ATR-FTIR technique at a 1 mm pixel resolution on the Infrared Microspectroscopy (IRM) Beamline at the Australian Synchrotron (Clayton, VIC, Australia), which was equipped with a VERTEX V80v FTIR spectrometer coupled to a Hyperion 3000 FTIR microscope (Bruker, Preston, VIC, Australia) and narrow-band mercury cadmium telluride (MCT) detector in the wavenumber range 3800–1000 cm^−1^ [29,30]. The thermal degradation profile of samples (under the nitrogen atmosphere) was studied using a TGA 8000^TM^ thermogravimetric analyzer (PerkinElmer Pty Ltd., Glen Waverley, VIC, Australia). The measurements were made at a nitrogen flow rate of 25 mL/h, and a heating rate of 10 °C/min and from 25 to 600 °C. The thermal characteristic of samples was investigated using a Discovery DSC 250 differential scanning calorimeter (TA Instruments, New Castle, DE, USA). The samples were sealed in an aluminum hermetic pans, and the measurements were performed in three cycles (heating–cooling–heating) under a nitrogen atmosphere (25 mL/h) with a heating rate of 10 °C/min from 25 to 350 °C. The rheological properties of the samples were analyzed using a Discovery HR-3 rheometer (TA Instruments, New Castle, DE, USA) equipped with a 25 mm diameter stainless steel parallel plate accessory, and temperature-controlled environmental chamber. The viscoelastic properties of the samples at 175 °C were measured as a function of oscillation strain (0.1–100%) and frequency (0.1–100 rad/s). The water sorption properties of the samples were tested according to ASTM D570 protocols, where the compression-molded samples were weighed, immersed in distilled water at 23 °C for 1 week, removed, dried with Kimwipes, and weighed. The sessile drop water contact angle of compression-molded samples was measured using a OCA20 contact angle measuring system (DataPhysics Instruments, Filderstadt, Germany), supported by SCA20 software (version 1.0). The surface roughness (as root mean square, RMS) of samples used for water contact angle measurement was measured using a P-16+ surface profiler (KLA-Tencor, Dresden, Germany), operated with an applied force of 1 mg, scan speed of 20 µm/sec, and sampling rate of 50 Hz. The tensile and flexural mechanical properties of the fabricated samples were obtained using an 5900R Universal Tester (Instron, Norwood, MA, USA) equipped with a 5 kN load cell. ASTM D638 and D790 protocols were followed for the tensile and flexural (three-point bend) tests. The tensile test was performed with a pull rate of 2 mm/min, whereas the flexural test was performed with a crosshead speed of 10 mm/min.

The degree of crystallinity (*X_C_*) of polypropylene in the rPP sample was calculated from its enthalpy of melting (∆*H_C_*) using the below equation:

(1) $$X_{C}=\frac{{∆H}_{C}}{{∆H}_{m}^{0}\;(1-m)}$$

where ∆*H_m_*^0^ is the enthalpy of melting of 100% crystalline polypropylene (207 J/g) [31], and *m* is the mass fraction of the maleic anhydride and wool fibers in the composite. In fiber-reinforced polymer composites, critical fiber length (*L_C_*), which is the minimum length of fibers required for effective load transfer from the polymer matrix to fiber filler, is an important parameter in improving the composite’s mechanical properties [32]. The *L_C_* of fabricated composites was calculated using the below equations:

(2) $$\tau_{i}=\frac{F_{max}}{2\pi rL}$$

(3) $$L_{C}=\frac{\sigma \times d}{2\times \tau_{i}}$$

where *τ_i_* is the interfacial shear strength, *F_max_* is the max debonding force, *r* is the radius of fiber, *L* is the length of the fiber, *σ* is the tenacity or ultimate tensile strength of the fiber, and *d* is the diameter of the fiber [32].

## 3. Results and Discussion

**Figure 2:**
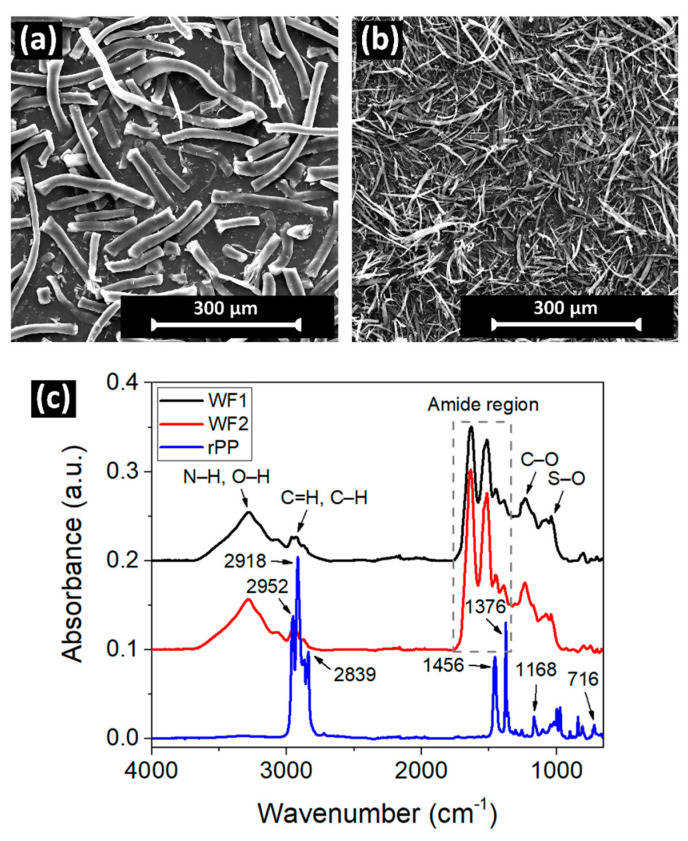
SEM images of wool fibers: (**a**) WF1, and (**b**) WF2. (**c**) ATR-FTIR spectra of wool fibers, and melt-mixed recycled polypropylene (rPP) sample.

Figure 2a,b show the SEM images (surface morphology) of wool fibers WF1 and WF2, respectively. The sample WF1 exhibited a fiber diameter and length in the range of 28–32 µm and 560–960 µm, respectively, which correspond to an aspect ratio (length/diameter) of ~25, calculated from the SEM images using the ImageJ software (Version 1.54). Conversely, the sample WF2 exhibited a fiber diameter and length in the range of 4–6 µm and 124–234 µm, respectively, which correspond to an aspect ratio (length/diameter) of ~35. For a given fiber weight, the number of fibers increases with a decrease in fiber diameter, so the specific surface area (area per unit weight) is inversely proportional to fiber diameter. Therefore, the specific surface area of WF2 > WF1, which can be clearly seen from the difference in volume of fibers in Figure 1. Here, the difference in the aspect ratio and specific surface area of wool fibers is anticipated to affect the polymer-wool interfacial surface area, which may influence the physicochemical properties of the composites, where the fibers provide the stiffness and strength, and the surrounding plastic matrix transfers the stress between fibers [33].

Figure 2c compares the ATR-FTIR spectra of the two wool fibers (WF1 and WF2) and melt-mixed rPP samples. The wool fibers exhibited FTIR absorptions in the wavenumber range of 3700–3000, 3000–2750, 1775–1575, 1575–1470, 1470–1320, 1280–1140, and 1140–960 cm^−1^, which can be attributed to an overlap of N–H and O–H regions, C=H and C–H regions, amide I (C=O region), amide II (N–H bond), amide III (C–N region), C–O region, S–O vibration, and C–H bond, respectively. These characteristic spectral features are in good agreement with literature reports on natural wool fibers [34]. Conversely, the melt-mixed rPP sample exhibited FTIR peaks at 2952, 2918, 2839, 1456, 1376, 1168, 998, 973, 841, and 809 cm^−1^, corresponding to the functional groups CH_3_ stretching, CH_2_ stretching, CH_3_ stretching, CH_3_ bending, CH_3_ bending, CH_3_ rocking and/or C–H waggling, CH_3_ rocking and/or C–C stretching, CH_3_ rocking and/or C–C stretching, CH_3_ and/or C–H rocking, and C–C stretching, respectively [35]. However, an additional FTIR band observed at 716 cm^−1^ suggested the presence of polyethylene contamination in the rPP sample. The polyethylene contamination of the rPP sample is part of the waste stream used. Therefore, based on the ratio of 1168/(1168 + 716) FTIR peaks, the percentage of polyethylene contamination in rPP was estimated as 7.0 ± 1.7 wt.% [36]. The polyethylene contamination was also confirmed by SEM and DSC analyses.

**Figure 3:**
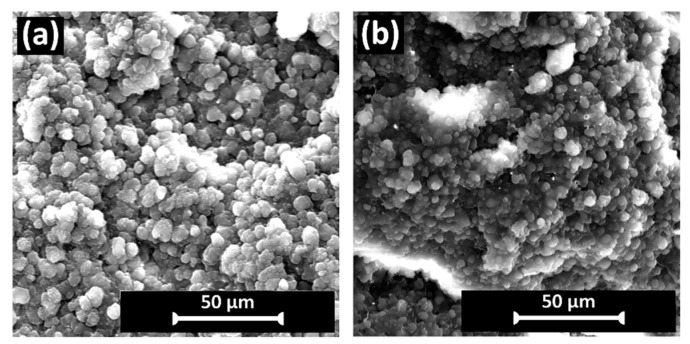
SEM images of the fracture surface morphology of melt-mixed (**a**) rPP, and (**b**) rPP with 5 wt.% polypropylene-graft-maleic anhydride compatibilizer (rPP-C) samples.

Figure 3a shows the SEM image of the fracture surface of the melt-mixed rPP sample, which exhibited an internal coalescent droplet (or spherical) morphology (~4 µm in size). This further supports the FTIR results, i.e., the presence of polyethylene contamination, where the viscosity ratio and interfacial tension between polypropylene and polyethylene melts have been reported to cause thermodynamic immiscibility in their blends, resulting in the formation of droplet structures (in this case, polyethylene) dispersed in a continuous matrix (rPP) during polymer processing [37]. The polyethylene droplet size and coalescence in the rPP matrix were observed to decrease (~2.5 µm in size) with the addition of 5 wt.% PP-g-MA as a compatibilizer (Figure 3b), which suggests improved interfacial interaction between the components, and is expected to influence the rheological and mechanical properties [38].

**Figure 4:**
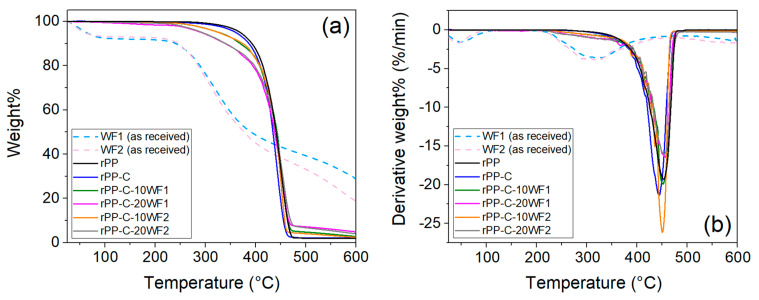
TGA thermograms: (**a**) weight loss profile, and (**b**) derivative curve of as-received wool fibers and fabricated composites.

The applications of polymer composites in automotive and building industries are largely based on their thermal stability, durability, and mechanical properties. Figure 4a,b show the TGA weight loss profile and the respective first derivative curve of the wool fibers and fabricated composites. The weight changes in polymeric materials can be caused by chemical reactions (e.g., oxidation and decomposition) as well as physical processes (e.g., desorption and sublimation) [39]. The obtained thermal properties such as the onset of degradation, first derivative peak temperature (point of greatest rate of change on the weight loss), and amount of char obtained are given in Table 2. The rPP sample exhibited an onset of degradation temperature of 364 °C, which systematically decreased with the addition of wool fibers, which can be attributed to the lower onset of degradation temperature of wool fibers (~230 °C). The wool fibers exhibited a first derivative peak degradation temperature of ~320 °C, and the rPP-C sample was slightly lower than the rPP sample (~452 °C). However, no significant change in the first derivative peak degradation temperature was observed for the fabricated composites compared to the rPP sample, which indicates its thermal stability. Moreover, no significant difference between composites comprising wool fibers WF1 and WF2 was measured. An increase in wool fiber content in the composites resulted in an increased amount of char (at 600 °C) because of the higher carbon content of wool fibers compared to PP.

**Figure 5:**
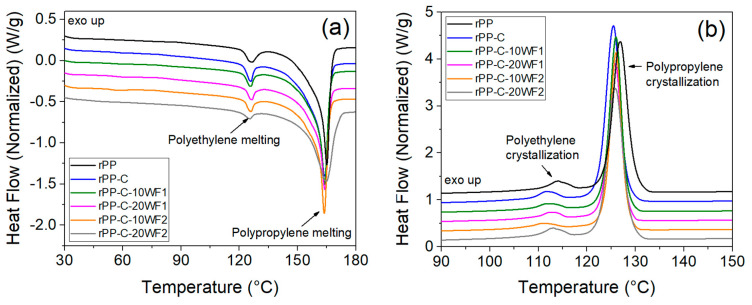
DSC thermograms: (**a**) heating and (**b**) cooling curves of fabricated composites.

**Figure 6:**
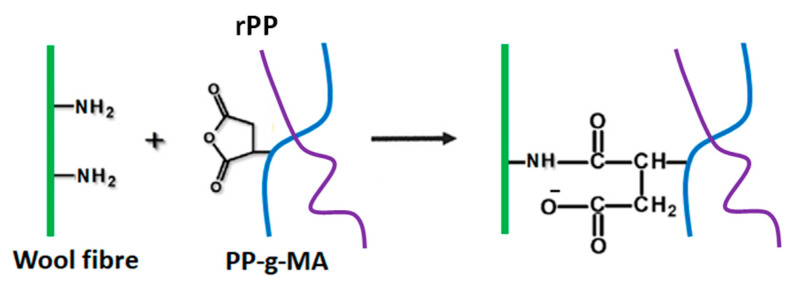
Schematic of interaction mechanism of polypropylene-graft-maleic anhydride on wool fibers in fabricated composites.

Thermal stability and mechanical properties of polymer composites largely depend on the degree of crystallinity of the polymer matrix. Figure 5a,b show the DSC curves of melting and crystallization events, respectively, of the polymer matrix of fabricated composites. The obtained thermal properties such as melting and crystallization temperature, and enthalpy, are given in Table 2. The endothermic peaks observed at ~164 °C and ~126 °C can be attributed to the melting and crystallization peaks of polypropylene [40]. Conversely, the peaks observed at ~126 °C and ~113 °C correspond to the melting and crystallization peaks of polyethylene [41]. This supports the FTIR and SEM results and further confirms the presence of noticeable polyethylene contamination in the rPP sample. The percentage of polyethylene contamination in rPP was estimated (using a calibration curve plotted from heats of fusion of the 100% pure polypropylene and polyethylene) as 7.4 wt.% from the measured DSC total heat of fusion (86.35 J/g), which verifies the estimation by FTIR results [42]. The rPP-C sample exhibited a slightly lower melting and crystallization peak temperature, which is due to the contribution of a relatively lower molecular weight of PP-g-MA compared to the rPP sample. This is also supported by the rheology results, discussed later. No significant difference in the polypropylene melting and crystallization temperature was observed for the fabricated composites. The slightly higher crystallinity obtained for the rPP-C sample compared to rPP further supports the relatively lower molecular weight of PP-g-MA [43]. The enthalpy of both melting and crystallization was observed to decrease with an increase in wool fiber content in the composites. However, the estimated crystallinity of rPP in composites was observed to increase with an increase in wool fiber content, i.e., from 36.35% to 37.32% for the rPP/WF1 composite, and 34.43% to 38.16% for rPP/WF2 composites (Table 2). The increase in crystallinity of rPP with the addition of wool fibers can be attributed to their intermolecular interaction, where the amine group of wool fibers can interact with maleic anhydride of PP-g-MA in the rPP matrix [44]. A schematic of the interaction mechanism of PP-g-MA on wool fibers is given in Figure 6.

**Figure 7:**
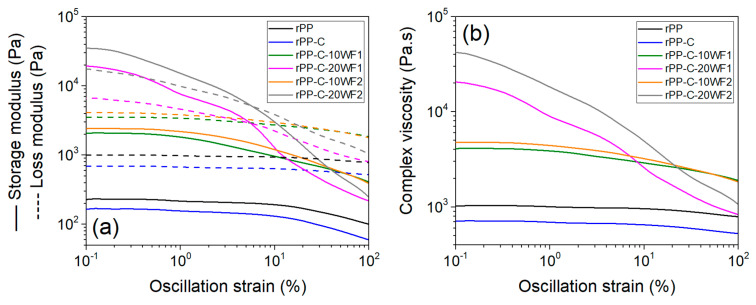
Dynamic rheology measurements: (**a**) storage (straight line) and loss (broken line) modulus, and (**b**) complex viscosity as function of oscillation strain.

Understanding the rheological behavior of polymer melts is essential for the effective material design and fabrication (polymer processing) of the composite. Figure 7a shows the viscoelastic properties of fabricated composites measured as a function of oscillation strain at the polymer processing (melt-mixing and compression molding) temperature used in this work. The storage modulus (G′) represents the ability of a material to store energy elastically, whereas the loss modulus (G″) represents the amount of energy dissipated in the sample when the material turns viscous [45]. At 175 °C, the rPP and rPP-C samples exhibited a storage modulus < loss modulus (i.e., liquid-like, or viscous behavior) for all tested oscillation strain values, and a linear viscoelastic region up to an oscillation strain (or critical strain) of 10%. The lower modulus values measured for the rPP-C sample can be attributed to the relatively less average molecular weight of the sample (contributed by relatively low molecular weight of PP-g-MA) compared to rPP [46], which further supports the DSC results. For thermoplastic composites, the critical strain value varies depending on filler (content, geometry, and dispersibility), and interfacial interactions [47]. The addition of wool fibers to the rPP matrix clearly increased the viscoelastic modulus values of the composites and decreased their linear viscoelastic region. Although the aspect ratio is different for WF1- and WF2-based composites, they exhibit similar rheology due to the fact that viscosity of the composites is governed by fiber melt interaction. Note that both fibers are below critical fiber length (discussed in detail later), and hence are short fibers.

The incorporation of fibers in the polymer system generally increases the viscosity and increases with fiber content. At a low concentration, the viscosity is expected to increase rapidly with an increasing concentration of the fibers because of the rapidly increasing collisions between fibers as they become packed more closely to each other. However, at a critical concentration level, random packing ceases and a further increase in fiber concentration leads to a more orderly anisotropic structure of the fibers in the melt, and these may now slide readily past one another, without contributing much to overall rheology. Composite melts with 20 wt.% wool fibers exhibited solid-like behavior (storage modulus < loss modulus) at low strain, whereas there was liquid-like behavior at high strains, which suggests increased fiber-entangled and interfacial interaction in the polymer matrix. This is further substantiated by the decrease trend in complex viscosity (a measure of the total resistance to flow) of composites measured with an increase in strain (Figure 7b)—shear thinning behavior—which is favorable for polymer processing techniques such as melt screw extrusion and 3D-printing [48,49].

**Figure 8:**
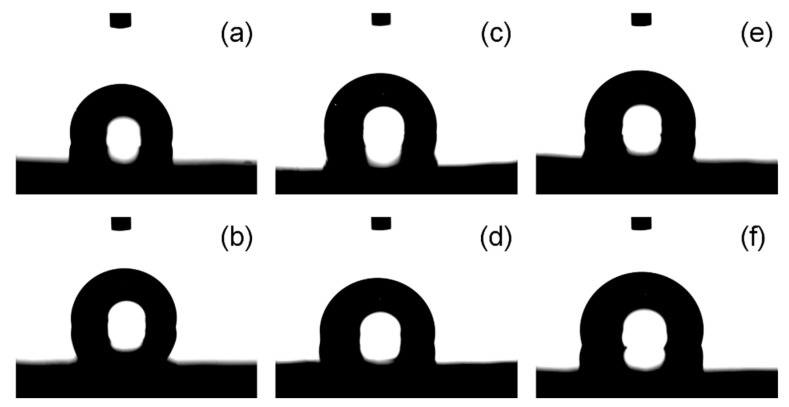
Contact angle measurement displaying water droplets on the surface of compression-molded samples: (**a**) rPP, (**b**) rPP-C, (**c**) rPP-C-10WF1, (**d**) rPP-C-20WF1, (**e**) rPP-C-10WF2, and (**f**) rPP-C-20WF2.

All the fabricated samples showed no increase in weight after being submerged in water for 1 week. Figure 8 shows the images of water droplets on the surface of fabricated samples. The measured values of the water contact angle and surface roughness of fabricated samples are given in Table 3. The rPP sample exhibited a water contact angle of 103.5 ± 0.4°, which slightly decreased with the addition of a compatibilizer (maleic anhydride), which can be attributed to a decrease in surface roughness from ~47 nm to ~36 nm. The incorporation of 10 wt.% wool fibers in the rPP matrix resulted in a decreased water contact angle, which can be attributed to a relatively lower water contact angle of untreated individual wool fibers (~78.2° [25]) compared to rPP-C. The rPP-C-10WF1 and rPP-C-10WF2 composites exhibited a water contact angle of 90.4 ± 2.1° and 95.1 ± 0.6°, respectively, and surface roughness of 31.1 ± 6.6 nm and 49.8 ± 3.5 nm, respectively. However, a further increase in wool fiber content to 20 wt.% resulted in an increased water contact angle of the composites, which may be due to an increase in surface roughness [50]. The observed difference between composites comprising WF1 and WF2 can be attributed to the difference in fiber diameter and surface roughness [51]. Therefore, the developed composites can be potentially used for outdoor applications.

**Figure 9:**
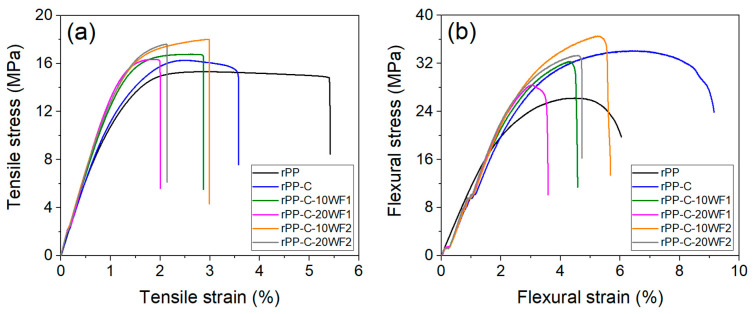
Mechanical property measurements: stress–strain curves of (**a**) tensile and (**b**) flexural test.

Figure 9a,b show the stress–strain plots of tensile and flexural moduli of the fabricated composite samples. The mechanical properties of samples obtained from the graphs are presented in Table 4. The 100% rPP sample used in this work exhibited tensile and flexural strength around 15.3 MPa and 26.2 MPa, respectively, which are in the lower range reported for rPP in the literature [52,53], and may be related to the amount of polyethylene and dry paint waste contamination. A noticeable increase in the Young’s modulus and tensile and flexural strength of rPP was observed with the addition of a compatibilizer (PP-g-MA), which can be attributed to improved interfacial interactions between PP and polyethylene contamination in the rPP matrix, which further supports the SEM results. The incorporation of WF1 (relatively low aspect ratio) into the rPP matrix showed no significant influence or improvement in the mechanical properties, whereas the incorporation of WF2 (relatively high aspect ratio) with an optimal fiber loading of 10 wt.% showed about an 18% increase in tensile strength and 39% increase in flexural strength. This is significantly higher than improvements reported in the literature for virgin PP/pre-consumer wool fiber composites [18,54,55,56], but relatively less compared to composites fabricated using silane (coupling agent) surface-treated wool fibers [20]. Moreover, this is the first work to report the use of both polypropylene and wool fibers as recycled materials in a composite. The developed composites could be used in the automotive industry, such as for interiors, and non-structural components [57]. This clearly demonstrates the potential of textile waste fibers in reinforcing rPP materials. However, increasing wool fiber content to 20 wt.% showed decreased trends in mechanical properties.

**Figure 10:**
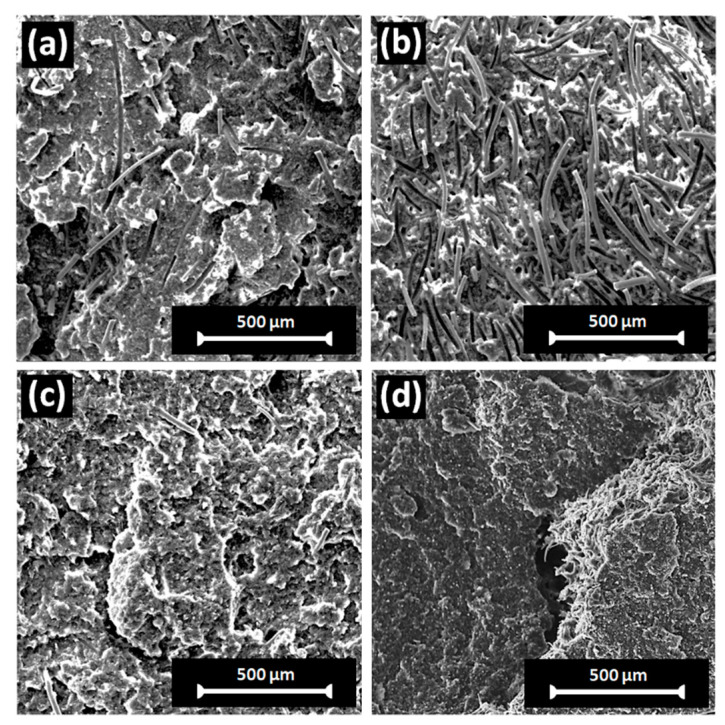
SEM images of the fracture surface of tensile-tested fabricated samples: (**a**) rPP-C-10WF1, (**b**) rPP-C-20WF1, (**c**) rPP-C-10WF2, and (**d**) rPP-C-20WF2.

The rheology and mechanical properties of fiber-reinforced composite materials are largely influenced by the aspect ratio, distribution, and orientation of fibers in the polymer matrix. Figure 10 shows an SEM image of the fracture surface morphology of tensile-tested fabricated samples. The composites exhibited a uniform distribution and random orientation of fibers in the droplet morphology of rPP-C. Moreover, at the fracture surface, the sockets of fibers after being pulled out and the fiber/matrix debonding can be clearly seen, suggesting that the main failure mechanism of the fabricated composite is interfacial debonding and fiber pull-out [58].

**Figure 11:**
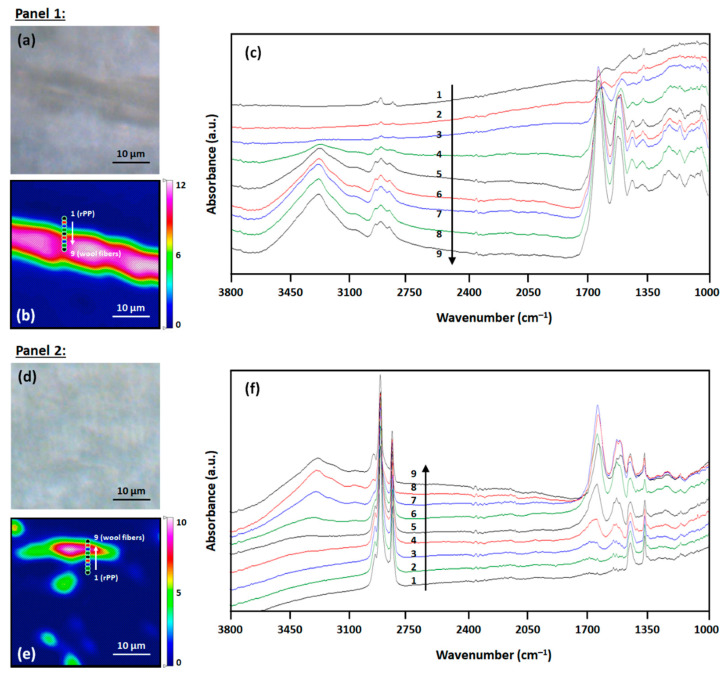
Synchrotron macro-ATR-FTIR results. Panel 1 (rPP-C-10WF1): (**a**) microscopic image, (**b**) chemical map of amide I band characteristic of WF1 in composite with colored markers demarcating line arrays across interfacial regions, and (**c**) comparison of individual spectra extracted from selected locations as indicated in panel (**b**). Panel 2 (rPP-C-10WF2): (**d**) microscopic image, (**e**) chemical map of amide I band characteristic of WF2 in composite with colored markers demarcating line arrays across interfacial regions, and (**f**) comparison of individual spectra extracted from selected locations as indicated in panel (**e**).

To gain further insights into their distributions and the interaction of the two components at the interface within the fabricated composites, rPP-C-10WF1 and rPP-C-10WF1 samples were subsequently analyzed using the high-resolution synchrotron macro-ATR-FTIR technique. This synchrotron-based chemical mapping technique provides 2D chemical images of specific chemical functionalities, and allows us to observe changes in spectral patterns across the interface to determine their interfacial interactions with surrounding materials, which can be correlated with other physical properties [59]. Figure 11 displays synchrotron macro-ATR-FTIR results obtained from microtomed cross-sections of the rPP-C-10WF1 (Panel 1) and rPP-C-10WF2 (Panel 2) composites. In this study, the amide I band at 1675 cm^−1^ and C-H bending mode of methylene groups at 1376 cm^−1^ were used as characteristic absorptions of the wool fibers and rPP, respectively. Figure 11b,e present the chemical maps generated by integrating area under the amide I band at 1675 cm^−1^. These heat maps reveal distinct distributions and phase domains of the wool fibers embedded within the two composites, which cannot be clearly identified in the corresponding microscopic images (Figure 11a,d). Specifically, the synchrotron macro-ATR-FTIR chemical images of the wool fibers presented here for the two composites agree well with the SEM images shown in Figure 2 and Figure 10, confirming that the rPP-C-10WF1 composite contains much larger wool fibers compared to those in the rPP-C-10WF2 sample. Furthermore, Figure 11c,f illustrate the gradual changes in the spectral pattern at the interface between the rPP polymer matrix and the embedded wool fibers (as labeled from 1 to 9). In both cases, the ν(O–H) stretching band observed in the range of 3500–3000 cm^−1^ shows progressive variations in band shape and peak position, suggesting alterations in the hydrogen-bonded characteristics across the interface [29]. Based on these results, hydrogen bonding interactions may play a significant role in the strength of the composites.

The interfacial shear strengths of the fabricated composites comprising WF1 and WF2 were calculated (using Equation (2)) in the range of 5–10 MPa and 111–340 MPa, respectively. Therefore, the relatively higher mechanical strength measured for the rPP/WF2 compared to rPP/WF1 composites can be attributed to its higher fiber-matrix interfacial shear strength (which is related to smaller diameter of WF2, i.e., higher interfacial surface area). The critical fiber length (*L_C_*) values of rPP/WF1 and rPP/WF2 composites were estimated in the range of 332–354 µm and 113–122 µm, respectively. In general, for fiber-reinforced composites, if the length of the fibers (*L*) >> *L_C_* (normal: *L* > 15 *L_C_* for effective fiber reinforcement), they are called continuous fibers, whereas fibers with *L* < 15 *L_C_* are called discontinuous. The fabricated rPP/WF1 and rPP/WF2 composites exhibited an *L*/*L_C_* ratio of ~2.5 and ~3.6, respectively. Therefore, the samples fabricated in this work are examples of discontinuous short fiber composites, where wool fibers were distributed randomly in a continuous rPP matrix.

## 4. Conclusions

In this work, we have systematically developed rPP/wool fiber composites using post-consumer waste products, such as paint pail plastic and biofiber textiles (with fibers of different aspect ratio). Composites fabricated by melt processing and using PP-g-MA as a compatibilizer exhibited coalescent micro-droplet morphology due to polyethylene and dry paint waste constituents in the r-PP. Composites fabricated with larger-aspect-ratio fibers exhibited superior rheological and mechanical properties compared to rPP, which can be attributed to a smaller diameter and distribution/entanglement of the fibers in the polymer matrix. This study demonstrates the compatibility of recycled and waste biofiber materials in a composite structure for an improved interface and better mechanical performance, which generally pose major scientific challenges. It also provides a sustainable platform to transform plastic waste into value-added products, suitable for auto parts, furniture, decking, and housing applications, which could generate new circular economy opportunities.

## Figures and Tables

**Table 1:** Compositions of fabricated samples.

Samples		Composition (wt.%)		
	rPP	WF1	WF2	PP-g-MA
rPP	100	-	-	-
rPP-C	95	-	-	5
rPP-C-10WF1	85	10	-	5
rPP-C-20WF1	75	20	-	5
rPP-C-10WF2	85	-	10	5
rPP-C-20WF2	75	-	20	5

**Table 2:** Thermal properties of fabricated composites measured by TGA and DSC techniques.

Samples	TGA Data			DSC Data				
	Onset of Degradation (°C)	First Derivative Peak Temperature (°C)	Char at 600 °C (wt.%)	Heating			Cooling	
				Melting Temperature of PP (°C)	Enthalpy of PP Melting (J/g)	Crystallinity of PP (%)	Crystallization Temperature of PP (°C)	Enthalpy of PP Crystallization (J/g)
rPP	364.21	452.58	1.99	165.02	66.75	32.25	126.88	68.31
rPP-C	359.56	444.26	2.03	164.03	70.87	34.41	125.47	76.24
rPP-C-10WF1	257.59	451.85	2.76	163.98	67.34	36.35	125.98	72.96
rPP-C-20WF1	223.25	453.51	4.82	163.85	61.42	37.32	125.84	68.47
rPP-C-10WF2	257.58	451.01	2.44	163.74	63.79	34.43	125.97	71.53
rPP-C-20WF2	223.23	454.36	4.09	164.31	62.79	38.16	125.86	62.01

**Table 3:** Water contact angle and surface roughness of fabricated composites.

Samples	Water Contact Angle (°)	RMS (nm)
rPP	103.5 ± 0.4	46.8 ± 3.7
rPP-C	100.1 ± 0.5	35.5 ± 4.5
rPP-C-10WF1	90.4 ± 2.1	31.1 ± 6.6
rPP-C-20WF1	101.2 ± 0.3	73.6 ± 7.5
rPP-C-10WF2	95.1 ± 0.6	49.8 ± 3.5
rPP-C-20WF2	107.4 ± 0.2	74.8 ± 7.9

**Table 4:** Mechanical properties of fabricated composites.

Samples	Tensile Properties			Flexural Properties		
	Young’s Modulus (GPa)	Tensile Strength (MPa)	Break Strain (%)	Young’s Modulus (GPa)	Flexural Strength (MPa)	Strain (%) at Maximum Strength
rPP	1.4 ± 0.02	15.31 ± 0.18	5.41 ± 0.11	1.22 ± 0.02	26.20 ± 0.04	4.70 ± 0.10
rPP-C	1.5 ± 0.06	16.26 ± 0.24	3.58 ± 0.10	1.88 ± 0.01	34.11 ± 0.18	6.58 ± 0.30
rPP-C-10WF1	1.6 ± 0.04	16.75 ± 0.81	2.87 ± 0.17	2.08 ± 0.06	32.28 ± 0.72	4.34 ± 0.20
rPP-C-20WF1	1.6 ± 0.03	16.34 ± 0.72	2.00 ± 0.13	2.05 ± 0.14	28.41 ± 0.93	3.06 ± 0.21
rPP-C-10WF2	1.6 ± 0.05	18.02 ± 1.06	2.98 ± 0.14	2.03 ± 0.05	36.54 ± 1.11	5.31 ± 0.25
rPP-C-20WF2	1.6 ± 0.06	17.59 ± 1.03	2.14 ± 0.16	2.05 ± 0.09	33.35 ± 1.43	4.58 ± 0.12

## Data Availability

Data is contained within the article.
